# Treatment of insulin resistance with tirzepatide leading to improvement of hair loss

**DOI:** 10.1016/j.jdcr.2024.06.001

**Published:** 2024-06-11

**Authors:** Emily R. Gordon, Sarah Musleh, Lindsey A. Bordone

**Affiliations:** a Columbia University Vagelos College of Physicians and Surgeons, New York, New York; b Anzara Health, New York, New York; c Department of Dermatology, Columbia University Irving Medical Center, New York, New York

**Keywords:** androgenic alopecia, GLP-1 agonist, insulin resistance, metabolic syndrome, tirzepatide

## Introduction

Insulin resistance, metabolic syndrome, and obesity have been linked to hair loss, although controversy remains about the relationship between these conditions.^1^ As increasing therapeutic options emerge for treatment of metabolic conditions including insulin resistance, their impact on hair loss may provide insight into pathogenesis of androgenic alopecia and its treatment. Tirzepatide is a novel glucose-dependent insulinotropic polypeptide and glucagon-like peptide-1 (GLP-1) receptor agonist for management of obesity and blood sugar levels in adults with type 2 diabetes; however, its impacts on alopecia are unknown. We present a 57-year-old man with androgenic alopecia who was treated with tirzepatide monotherapy and had significant improvement in hair density within 6 months, alongside improvement in insulin resistance and weight loss. This report provides an example of how treating insulin resistance, specifically with GLP-1 agonists, may also improve hair loss for patients with metabolic syndrome.

## Case report

**Fig 1:**
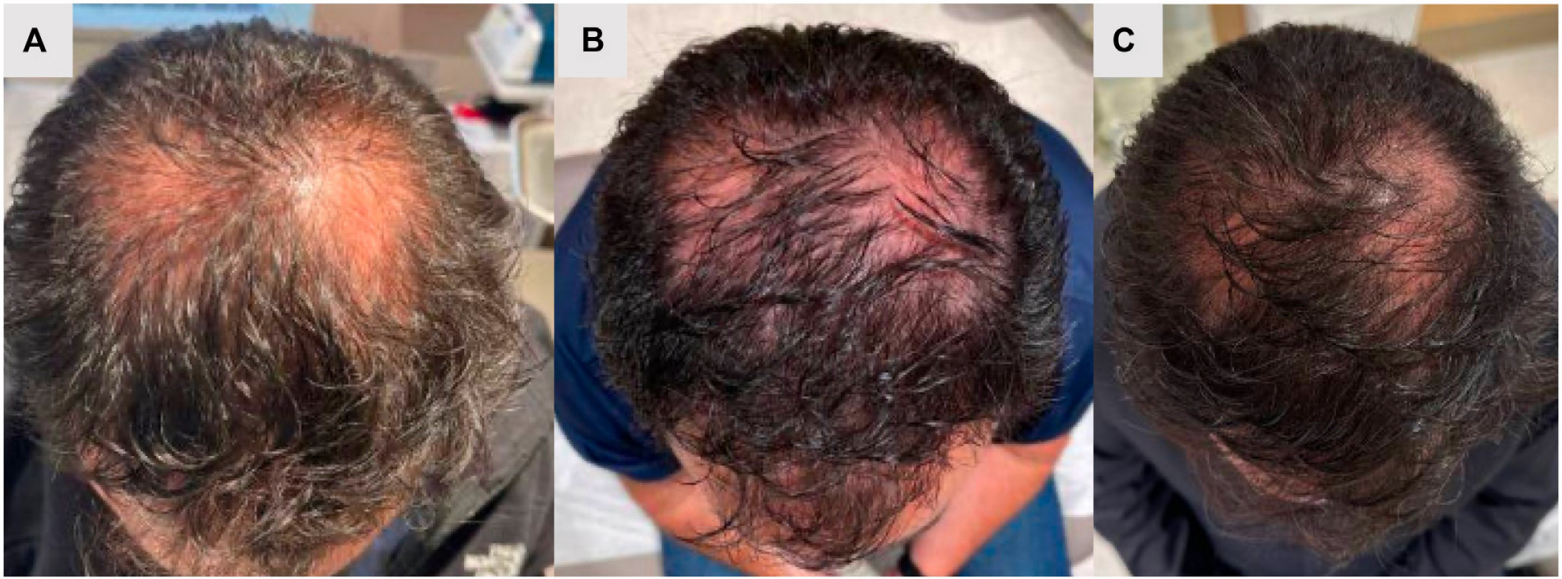
Androgenic alopecia with improvement after treatment with tirzepatide. Clinical images at **(A)** initial presentation, **(B)** after 6 months of tirzepatide monotherapy, and **(C)** after 1 year of tirzepatide monotherapy.

A 57-year-old man presented for evaluation of 1 year of hair loss. His body mass index was 33.45 kg/m^2^, and he denied recent illnesses, other possible precipitators of telogen effluvium, and history of medical conditions including diabetes, thyroid disease, or lupus erythematosus. Physical examination showed increased hair shedding, vertex scalp with decreased hair density and widened part, bitemporal scalp with mild hair recession, negative hair pull test. Trichoscopic examination demonstrated hair thickness heterogeneity, yellow dots, vellus hairs, and perifollicular hyperpigmentation, consistent with androgenic alopecia (Fig 1, *A*). He had velvety hyperpigmented plaques on the neck consistent with acanthosis nigricans. His bloodwork revealed hemoglobin A1C of 5 (normal, <5.7), C-peptide 2.14 (normal, 0.8-1.8), fasting insulin 13.1 (normal, 5-12), and fasting glucose 99 (normal, 70-100), with insulin resistance score 3.2 (normal, <2), indicative of insulin resistance. He was referred to endocrinology for evaluation of insulin resistance where he was started on tirzepatide monotherapy, 2.5 mg every 7 days. He continued this dose for 3 months and the dose was then increased to 5 mg weekly. Within 6 months of tirzepatide initiation, he experienced weight loss of 20 pounds and significant improvement in hair density (Fig 1, *B*). He remained on 5 mg weekly for 6 months before moving to his final dose of 7.5 mg weekly which he has been on since. After 1 year of tirzepatide monotherapy, he had 30 pounds of weight loss and additional hair regrowth (Fig 1, *C*). Of note, the patient was not using other medications for hair growth. His subsequent A1C was 5.5, C-peptide 1.67, fasting insulin 8, and fasting glucose 87, reflective of insulin resistance score 1.7, within normal limits.

## Discussion

The association between insulin resistance and hyperandrogenism-related conditions such as acne and androgenic alopecia remains controversial.^2^ Several case-control studies failed to demonstrate statistically significant differences in insulin resistance between patients with androgenic alopecia and age-matched controls. Specifically, Nabaie et al^3^ found no difference in serum fasting insulin level, fasting blood glucose, serum total cholesterol, triglyceride, high-density lipoprotein, and insulin resistance between 97 cases and 87 controls. Ozbas Gok et al^4^ included 74 patients with androgenic alopecia with no known history of glucose metabolism disease, and compared them to 42 age-matched controls, finding no association between alopecia and metabolic syndrome or insulin resistance. However, they excluded elderly patients and most patients had mild alopecia compared with other studies. Swaroop et al^5^ also failed to identify a significant difference in insulin resistance between 50 patients with alopecia and 50 controls, however, they did find that patients with androgenic alopecia had significantly higher levels of fasting insulin and metabolic syndrome.

In contrast, other studies established clear connections between insulin resistance and androgenic alopecia. Bakry et al^1^ compared 100 patients with androgenetic alopecia to 100 controls, and Acibucu et al^6^ evaluated 80 patients with androgenic alopecia versus 48 controls. Both studies found that metabolic syndrome and insulin resistance were significantly higher in patients with androgenic alopecia, with specifically elevated body mass index, blood pressure, fasting glucose, high-density lipoprotein, and fasting insulin among patients with alopecia. González-González et al^7^ and Mumcuoglu et al^8^ also found homeostatic metabolic assessment for insulin resistance and fasting insulin resistance index were higher in men with alopecia compared with age-matched controls. Thus, it was proposed that androgenic alopecia may serve as a marker of insulin resistance.^9^

Prior studies are largely correlative and do not investigate the mechanistic relationship between androgenic alopecia and insulin resistance. Researchers have not studied how treatment of insulin resistance impacts hair loss. We describe the first case of a patient with androgenic alopecia, who was treated for insulin resistance with tirzepatide monotherapy, with significant increase in hair density within 6 months, along with weight loss and improvement in insulin resistance.

Androgenic alopecia is suspected to arise from overstimulation of the androgen hormone cascade in patients with genetic predisposition, leading to miniaturization of the dermal papilla of hair follicles. Dihydrotestosterone (DHT) can be increasingly produced in an overactive androgen state, inhibiting cell mitosis in dermal papilla and contributing to apoptosis.^3^ Insulin may contribute to androgenic alopecia through vasoconstriction and nutrient deficiency, along with enhancement of the effects of testosterone.^1^ Insulin resistance is associated with vasoactive substances that lead to endothelial dysfunction and cause microcirculatory disturbance, perifollicular vasoconstriction, and proliferation of smooth muscle cells in vascular walls. The result is microvascular insufficiency, local-tissue hypoxia, and increasing miniaturization of hair follicles.^1^ Moreover, insulin can increase DHT levels *de novo* from cholesterol or through conversion of testosterone to DHT.^3^ Insulin has been shown to increase DHT levels through activation of 5-α-reductase activity in patients with obesity.^4^ These various mechanisms demonstrate how insulin resistance, leading to increased insulin levels, may contribute to miniaturization of hair follicles, worsening androgenic alopecia.

Tirzepatide is a novel glucose-dependent insulinotropic polypeptide and GLP-1 agonist with substantial benefits in patients with type 2 diabetes and obesity. Although some reports link GLP-1 agonists to hair loss, this effect appears temporary and due to telogen effluvium secondary to weight loss.^10^ Treatment with tirzepatide has demonstrated significant and rapid improvement in glycemic control through concurrent improvements in beta-cell function and insulin sensitivity. It is possible that by minimizing insulin resistance, tirzepatide avoids the pathogenic miniaturization of the hair follicle responsible for alopecia. Additional investigation into the effects of GLP-1 agonists and treatments of insulin resistance on alopecia are needed, as these therapies may both lead to improvement in metabolic syndrome and their sequelae including hair loss.

## Conflicts of interest

None disclosed.
